# Ultrafast laser ablation, intrinsic threshold, and nanopatterning of monolayer molybdenum disulfide

**DOI:** 10.1038/s41598-022-10820-w

**Published:** 2022-04-28

**Authors:** Joel M. Solomon, Sabeeh Irfan Ahmad, Arpit Dave, Li-Syuan Lu, Fatemeh HadavandMirzaee, Shih-Chu Lin, Sih-Hua Chen, Chih-Wei Luo, Wen-Hao Chang, Tsing-Hua Her

**Affiliations:** 1 Department of Physics and Optical Science, The University of North Carolina at Charlotte, Charlotte, NC 28223 USA; 2 Department of Electrophysics, National Yang Ming Chiao Tung University, Hsinchu, 30010 Taiwan; 3 Research Center for Applied Sciences, Academia Sinica, Taipei, 11529 Taiwan; 4 Institute of Physics and Center for Emergent Functional Matter Science, National Yang Ming Chiao Tung University, Hsinchu, 30010 Taiwan; 5 National Synchrotron Radiation Research Center (NSRRC), Hsinchu, 30076 Taiwan

**Keywords:** Surfaces, interfaces and thin films, Two-dimensional materials, Two-dimensional materials, Nonlinear optics, Graphene, Two-dimensional materials, Surface patterning, Ultrafast lasers, Nonlinear optics, Laser material processing, Synthesis and processing, Surfaces, interfaces and thin films, Two-dimensional materials, Surface patterning, Nonlinear optics

## Abstract

Laser direct writing is an attractive method for patterning 2D materials without contamination. Literature shows that the ultrafast ablation threshold of graphene across substrates varies by an order of magnitude. Some attribute it to the thermal coupling to the substrates, but it remains by and large an open question. For the first time the effect of substrates on the femtosecond ablation of 2D materials is studied using MoS_2_ as an example. We show unambiguously that femtosecond ablation of MoS_2_ is an adiabatic process with negligible heat transfer to the substrates. The observed threshold variation is due to the etalon effect which was not identified before for the laser ablation of 2D materials. Subsequently, an intrinsic ablation threshold is proposed as a true threshold parameter for 2D materials. Additionally, we demonstrate for the first time femtosecond laser patterning of monolayer MoS_2_ with sub-micron resolution and mm/s speed. Moreover, engineered substrates are shown to enhance the ablation efficiency, enabling patterning with low-power ultrafast oscillators. Finally, a zero-thickness approximation is introduced to predict the field enhancement with simple analytical expressions. Our work clarifies the role of substrates on ablation and firmly establishes ultrafast laser ablation as a viable route to pattern 2D materials.

## Introduction

Single atomic layer materials such as graphene, transition metal dichalcogenides (TMDs), and hexagonal boron nitride have been studied extensively for their novel electronic and optical properties^1,2^. Graphene exhibits strong wavelength-independent absorption of 2.3%^1^ and high carrier mobilities reaching 200,000 cm^2^/(V∙s) if extrinsic disorder is eliminated^3^. TMDs such as Molybdenum disulfide (MoS_2_) and Tungsten disulfide (WS_2_) are of great interest because of their transition from indirect to direct band gap and strong excitonic resonances at room temperature as the number of layers is reduced to a monolayer^4,5^. Both graphene and MoS_2_ have demonstrated phenomenal mechanical robustness^6,7^ and optical stability under intense femtosecond excitation^8,9^. These properties have led to the research and development of 2D material-based electronic and optoelectronic devices such as transistors^1,10^, photodetectors^1,11^, and additional heterostructure devices^12^.

For such device applications, reliable patterning techniques are essential to selectively remove 2D materials for specific sizes and geometries. Although electron beam and photolithography have been used extensively to pattern 2D materials, they suffer from high costs, complexity, vacuum operation requirements, and more importantly are prone to leave behind contaminates or polymer residues, causing damage or unwanted doping which can inadvertently degrade their electrical properties^13^. In this regard, laser ablation is a promising technique to pattern 2D materials that is in situ, resist-free, and maskless. Specifically, the ultrafast laser ablation and patterning of graphene based on oxidative burning has been demonstrated on several substrates^14^, where scanning rates as high as tens of mm/s can be achieved with a laser fluence of a couple hundred mJ/cm^2^ from laser amplifiers^15^. In addition, sub-diffraction-limited ablated features under 100 nm can be obtained with shaped picosecond laser beams^14^. In contrast, little research has been conducted on the femtosecond ablation of monolayer TMDs. Paradisanos et al*.* has studied the multi-shot degradation of exfoliated monolayer and bulk MoS_2_ and reported single-shot ablation thresholds based on the appearance of submicron-sized distortion^16^. Pan, Y. et al*.* studied the laser-induced sub-wavelength ripple formation on a natural MoS_2_ crystal which they attributed to the interaction of spallation and sublimation of the crystal with the laser induced surface plasmon polaritons^17^. Similarly, Pan, C. et al*.* investigated ablation mechanisms of bulk MoS_2_ under intense femtosecond excitation and determined that the ablation was mediated by sublimation at weak pumping and melting at strong pumping^18^. Despite these efforts, a rigorous investigation of the threshold fluence and ultrafast laser patterning of monolayer TMDs has not been demonstrated. We note that continuous-wave (CW) 532 nm lasers have been demonstrated to sublimate monolayer MoS_2_ on a SiO_2_/Si substrate with a 200 nm spatial resolution^19^, though the patterning speed is slow due to its photothermal nature. The throughput, however, can be substantially increased using an optothermoplasmonic substrate which then requires transferring the patterned MoS_2_ film to other substrates^20^.

Since many applications require a supporting substrate, understanding its effect on the laser ablation of 2D materials is important. Although ultrafast laser ablation of graphene has been extensively studied, the role of the substrates is still not clear. The reported ablation thresholds from many studies made by similar pulse widths (~50–100 fs) and wavelengths (~800 nm) differ by one order of magnitude among suspended graphene and graphene supported by borosilicate glass, Al_2_O_3_, and 285 nm SiO_2_/Si substrates^15,21–25^. Surprisingly, such differences have never been discussed or understood. Beyond mechanical support, substrates have been routinely claimed to act as a heat sink to explain why CW laser thinning of multi-layer graphene and MoS_2_ self-terminates at monolayers^19,26^. Other groups also observed that the ablation threshold for both femtosecond and CW excitation are lower for suspended 2D materials than those supported on a SiO_2_/Si substrate, which was again attributed to heat dissipation through the supporting substrates^22,27^. Optically, substrates are known to enhance the light outcoupling of 2D materials through the etalon effect. For SiO_2_/Si substrates, the Raman scattering was shown to strongly depend on the SiO_2_ thickness for graphene^28^, which led to the optimization of both the Raman scattering and photoluminescence of WSe_2_ by controlling the SiO_2_ layer thickness where the largest enhancement occurred for a SiO_2_ thickness of about 90 nm for 532 nm excitation^29^. Similar enhancement for Raman scattering, photoluminescence, and second harmonic generation was obtained by using distributed Bragg reflectors (DBRs) as a substrate for MoS_2_^30^. Improved optical contrast of graphene and MoS_2_ was achieved by designing multilayer heterostructure substrates where an optical contrast of 430% was obtained for monolayer MoS_2_^31,32^. We note that the etalon effect has been previously shown to modulate the laser thinning efficiency of multilayer graphene^26^, but has never been studied for the laser ablation of 2D materials.

In this work, we studied the femtosecond laser ablation of monolayer MoS_2_ on a variety of common substrates. Notably, we demonstrated this process is both high speed (~5 mm/s) and high resolution (~250 nm with a 0.55 NA objective at 800 nm). Moreover, the influence of substrates on the ablation threshold fluence $F_{th}$ was investigated, both in single-shot and line-scan modes. It was shown that the femtosecond laser ablation of transferred monolayer MoS_2_ is adiabatic where the heat dissipation through the supporting substrates is negligible, and the variation in $F_{th}$ among substrates can be largely explained by the substrates’ etalon effect. Based on our finding, an all-dielectric DBR substrate was realized to reduce $F_{th}$ by 7× compared to that of sapphire to enable laser pattering using low-power femtosecond oscillators. Furthermore, we introduced an intrinsic ablation threshold fluence $F_{th}^{int}$ as a substrate-independent threshold parameter for the laser ablation of 2D materials. We also introduced the zero-thickness approximation to substantially simplify the calculation of the etalon effect for laser ablation. Combined with the knowledge of $F_{th}^{int}$, this makes the incident $F_{th}$ on any substrate predictable. Our work clarifies the role of substrates and provides a foundation for rapid prototyping of 2D-material devices using femtosecond laser ablation.

## Results and discussion

### Zero-thickness approximation

Previous studies on the etalon effect of monolayer 2D materials focused on engineering the Raman scattering, photoluminescence, and second-harmonic generation by optimizing the internal field at the excitation wavelength and the outcoupling efficiency at the emission wavelength^28–30^. As a result, the theoretical enhancement can only be calculated computationally. For ablation, only the excitation enhancement is of concern and we show below that the internal field ${\mathcal{E}}_{2DM}$ at the excitation wavelength has a simple analytical approximation. The substrates used in this study include sapphire (Al_2_O_3_), borosilicate glass, 70 nm thick gold (Au) film on a glass substrate, 90 nm SiO_2_/Si, and two custom designed DBR substrates: one DBR substrate (DBR800(+)) targets maximal intensity enhancement and the other (DBR800(-)) targets maximal intensity suppression. The system can be modeled as an asymmetric etalon composed of air, a 2D material, and a substrate (Supplementary Fig. S1a). If the effective reflection coefficient between the monolayer and the substrate $\tilde{r}_{1s} = r_{o} \exp \left( {i\phi } \right)$ is known, then the spatial distribution of the electric field inside the monolayer ${\mathcal{E}}_{2DM} \left( x \right)$ can be rigorously calculated using the Airy formula (Supplementary Eq. (S1)). Since monolayer 2D materials are much thinner compared to the wavelength investigated here, we introduce the zero-thickness approximation (ZTA), to simplify the internal field ${\mathcal{E}}_{2DM} \left( x \right)$ from Supplementary Eq. (S1) to become

1 $${\mathcal{E}}_{2DM} \left( x \right) \approx {\mathcal{E}}_{2DM}^{ZTA} = {\mathcal{E}}_{inc} \tilde{t}_{01} \left( {\frac{{1 + \tilde{r}_{1s} }}{{1 - \tilde{r}_{1s} \tilde{r}_{10} }}} \right),$$

where ${\mathcal{E}}_{inc}$ is the incident electric field and $\tilde{t}_{ij}$ and $\tilde{r}_{ij}$ are Fresnel transmission and reflection coefficients from the *i*^th^ to *j*^th^ medium, respectively. For single-material substrates such as Al_2_O_3_, glass, or a thick Au film, $\tilde{r}_{1s}$ is simply the Fresnel reflection coefficient (Supplementary Eq. (S3)), and the internal field ${\mathcal{E}}_{2DM}$ becomes approximately

2 $${\mathcal{E}}_{2DM}^{ZTA} = {\mathcal{E}}_{inc} \left( {\frac{2}{{1 + \tilde{n}_{s} }}} \right),$$

where $\tilde{n}_{s}$ is the complex refractive index of the substrate. For SiO_2_/Si substrates with a silica layer thickness of $d_{2}$, $\tilde{r}_{1s}$ can be calculated analytically using an asymmetric etalon composed of a TMD, SiO_2,_ and Si (Supplementary Eq. (S4)), and ${\mathcal{E}}_{2DM}$ becomes approximately

3 $${\mathcal{E}}_{2DM}^{ZTA} = {\mathcal{E}}_{inc} \left( {\frac{{2\left[ {\left( {\tilde{n}_{2} + \tilde{n}_{s} } \right) - \left( {\tilde{n}_{s} - \tilde{n}_{2} } \right)e^{{i2\beta_{2} d_{2} }} } \right]}}{{\left( {1 + \tilde{n}_{2} } \right)\left( {\tilde{n}_{2} + \tilde{n}_{s} } \right) + \left( {\tilde{n}_{2} - 1} \right)\left( {\tilde{n}_{s} - \tilde{n}_{2} } \right)e^{{i2\beta_{2} d_{2} }} }}} \right),$$

where $\beta_{2} = 2\pi \tilde{n}_{2} /\lambda_{0}$, and $\tilde{n}_{2}$ and $\tilde{n}_{s}$ are the refractive indices of SiO_2_ and Si, respectively. For DBR substrates, an analytical expression of ${\mathcal{E}}_{2DM}^{ZTA}$ can be found in Supplementary Eq. (S12). Details about the DBR design and fabrication can also be found in Supplementary Fig. S5. Equations (2)-(3) and Supplementary Eq. (S12) clearly show ${\mathcal{E}}_{2DM}^{ZTA}$ is independent of the 2D material. In fact, this result can be extended to arbitrary stratified substrates with the proof being presented in Supplementary Eq. (S9).

**Figure 1:**
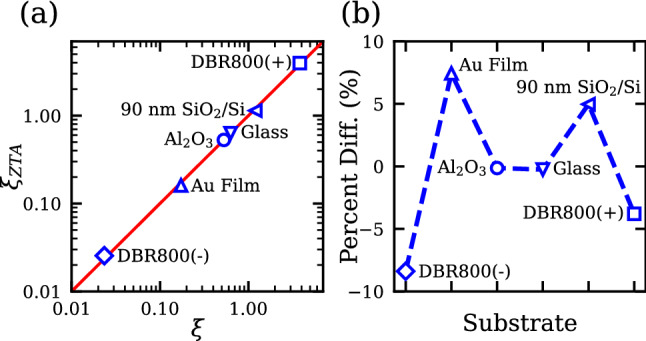
(**a**) Comparison of the internal intensity enhancement factor calculated from the rigorous Airy formula $\xi$ and ZTA $\xi_{ZTA}$ at 800 nm. The red line represents the ideal one-to-one ratio. (**b**) The percent difference between $\xi$ and $\xi_{ZTA}$ for the substrates in (**a**). A positive percentage means $\xi$ is larger than $\xi_{ZTA}$.

For a given substrate, we can define an internal intensity enhancement factor $\xi = \left| {{\mathcal{E}}_{2DM} } \right|^{2} /\left| {{\mathcal{E}}_{inc} } \right|^{2}$. Figure 1a compares the $\xi_{ZTA}$ calculated from Eqs. (2), (3), and (S9) with the rigorous $\xi$ calculated from Supplementary Eq. (S1). For the latter, the intensity is averaged over the thickness of the 2D material according to Supplementary Eq. (S2). Figure 1a shows excellent agreement between $\xi$ and $\xi_{ZTA}$ for the various substrates under consideration. The red line in Fig. 1a represents the ideal one-to-one ratio. Interestingly, the 90 nm SiO_2_/Si substrate has a $\xi$ close to unity (~1.14). Figure 1b shows that the percent differences for various substrates are all within 5% except the Au film (~7.4%) and the DBR800(-) substrate (~ − 8.4%). For the former, the large difference is due to Au’s large extinction coefficient (~5 at 800 nm), while for the latter the DBR800(-) substrate simply has a predicted internal intensity close to zero. As shown in the Supplementary Information, the ZTA remains valid for 2D materials consisting of a few layers. Specifically, the ZTA starts to deviate by ~10% when the number of layers is greater than seven for MoS_2_ supported by an Al_2_O_3_ substrate. As supported by Fig. 1, the excellent agreement between $\xi$ and $\xi_{ZTA}$ indicates the internal field ${\mathcal{E}}_{2DM}$ inside the monolayer 2D material is to a very good approximation solely determined by the surrounding media. The result is believed to be very useful for practical applications as $\xi_{ZTA}$ can be applied to all 2D materials.

### Intrinsic ablation threshold

**Figure 2:**
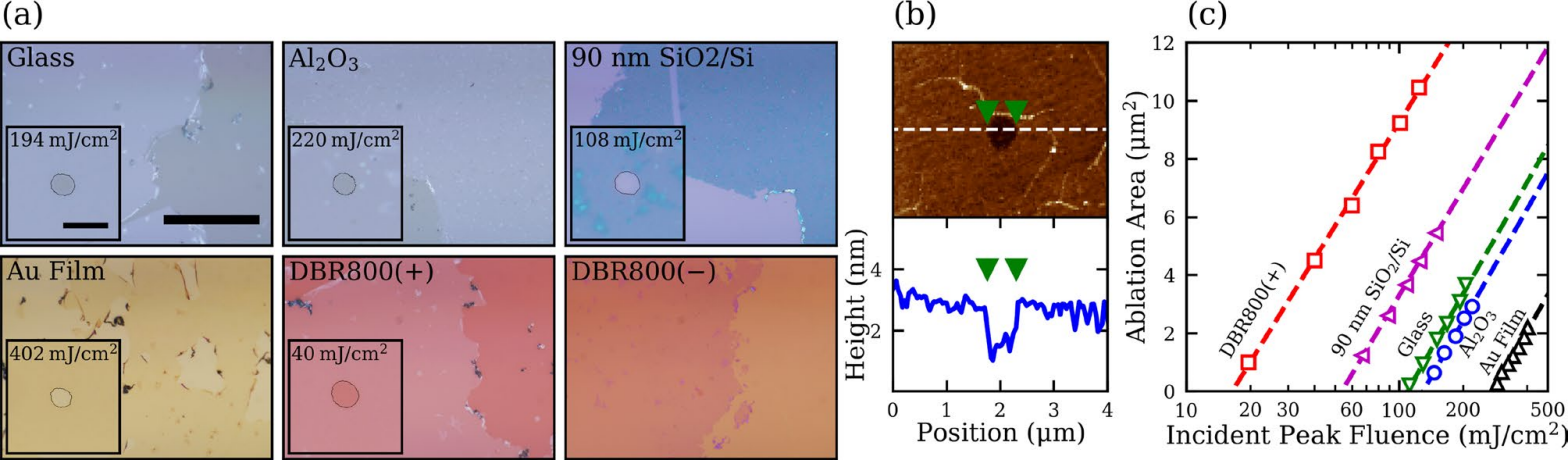
(**a**) Optical images of monolayer MoS_2_ films on different substrates, demonstrating the variation in optical contrast. The scale bar is 50 μm. The inset images show ablated holes of similar ablation areas at the indicated laser fluence. The contour of these holes is outlined. The scale bar of the inset images is 4 μm. (**b**) AFM scan and its cross-sectional profile of a typical ablated hole of MoS_2_ on Al_2_O_3_. (**c**) The ablation areas as a function of the peak fluence of the incident pulse. The intercept of the fit with the horizontal axis represents the ablation threshold, and the slope is proportional to the laser spot size.

To experimentally investigate this etalon effect in the ultrafast laser ablation of 2D materials, monolayer MoS_2_ is used since it is one of the most widely studied TMDs, but the results here are expected to apply for all 2D materials in general. As outlined in the Materials and Methods section, monolayer MoS_2_ films were CVD-grown on Al_2_O_3_ substrates and transferred to all the substrates used in this work (Fig. 2a). A single pulse from an ultrafast amplifier operated at 160 fs and 800 nm was focused to a spot radius of 1.9 µm on the MoS_2_ film using a 10× microscope objective with a 0.26 NA. The sample was translated to a fresh spot for subsequent exposures to avoid incubation effects. Figure 2a shows optical images of transferred monolayer MoS_2_ films on various substrates where single-shot ablated holes with similar diameters are shown in the insets. The fluences ranged from 20 mJ/cm^2^ to 400 mJ/cm^2^, and no ablation was observed for MoS_2_ on the DBR800(-) substrate before the substrate itself was damaged. Overall, Fig. 2a clearly demonstrates that substrates have a strong influence on the optical contrast of the films and on the ablation fluence required to make holes of similar size. Figure 2b shows an atomic force microscope (AFM) image and cross-sectional profile of a typical ablation spot in the MoS_2_ film on Al_2_O_3_, indicating that material has been removed. The depth of 1.2 nm for the ablated hole is the typical thickness of a transferred MoS_2_ monolayer as shown in Supplementary Fig. S4b, signifying that the underlying substrate remains undamaged.

Next, to accurately measure the ablation threshold, the ablation area was measured as a function of the pulse energy, following the method outlined by Liu^33^. Figure 2c shows the ablated area as a function of peak incident fluence for different substrates. The experimentally determined threshold fluences $F_{th}$ of MoS_2_ are approximately 130, 276, 110, 54, and 16 mJ/cm^2^, for the Al_2_O_3_, Au film, glass, 90 nm SiO_2_/Si, and DBR800(+) substrates, respectively. Figure 2c clearly shows that $F_{th}$ on various substrates taken by the same laser pulses can differ by an order of magnitude, confirming that the substrates have a very strong influence on the laser ablation of 2D materials. If the observed variation in $F_{th}$ is purely due to the etalon effect, $F_{th}$ should be inversely proportional to the internal intensity enhancement in the MoS_2_ monolayer, that is,

4 $$F_{th} \xi \approx F_{th} \xi_{ZTA} = {\text{constant}} = F_{th}^{{int}} .$$

Equation (4) defines the intrinsic ablation threshold $F_{th}^{int}$, which is the ablation threshold fluence for a free-standing 2D material where $\xi_{ZTA}$ equals unity (Eq. (2)). $F_{th}^{int}$ is a unique threshold parameter for a 2D material that is independent of the underlying substrate, corresponding to the energy required to remove atoms per unit area. By further defining a normalized ablation threshold $F_{th}^{\prime} = F_{th} /F_{th}^{int}$, Eq. (4) is reduced to a more compact form

5 $$F_{th}^{\prime} \xi_{ZTA} = 1.$$

**Figure 3:**
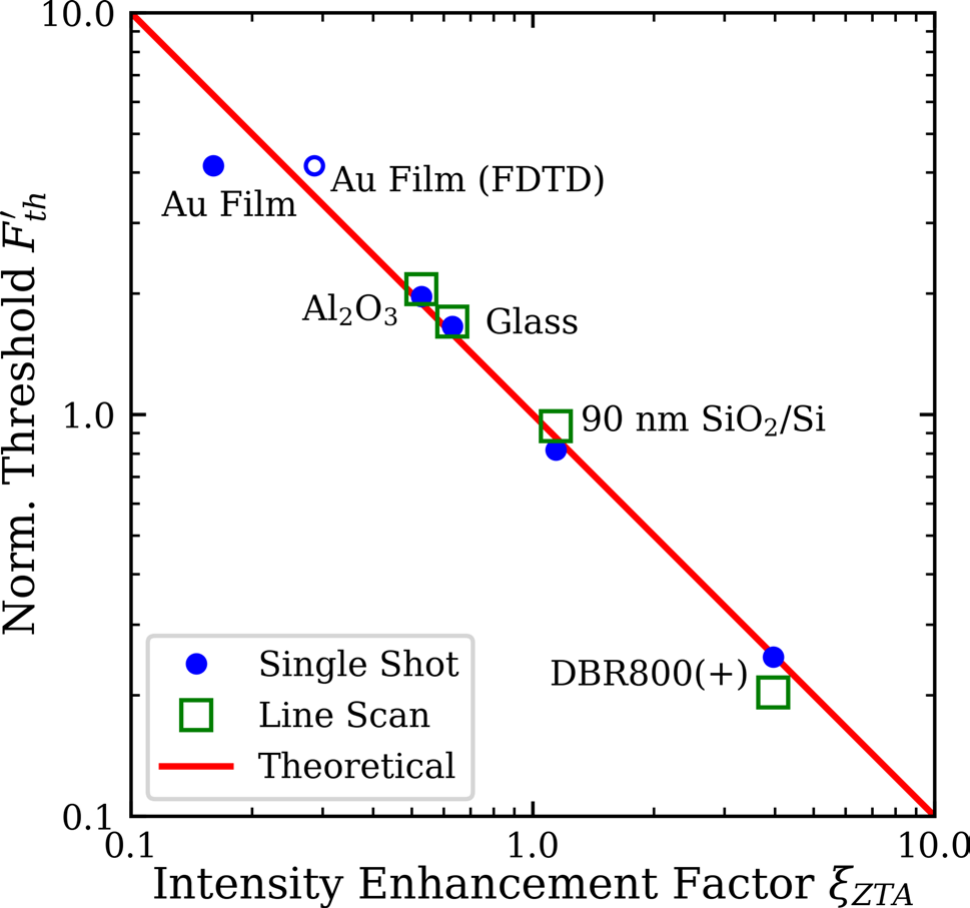
Scaling between the normalized ablation threshold and the calculated internal intensity enhancement factor at 800 nm for both single-shot and line-scan ablation. The internal intensity was calculated following the ZTA for all substrates. An additional point for the internal intensity for the Au film was calculated by FDTD. The ablation threshold is normalized to the intrinsic ablation threshold $F_{th}^{int}$.

The experimentally determined ablation thresholds for MoS_2_ supported by the Al_2_O_3_, glass, 90 nm SiO_2_/Si, and DBR800(+) substrates in Fig. 2c are fitted to $F_{th} = F_{th}^{int} /\xi_{ZTA}$ where $F_{th}^{int}$ is used as a fitting parameter (Supplementary Fig. S8). This fit yields $F_{th}^{int} \approx$ 66 mJ/cm^2^ for monolayer MoS_2_. $F_{th}^{\prime}$ and $\xi_{ZTA}$ for various substrates are shown as solid circles in Fig. 3, together with the theoretical line of Eq. (5). The excellent agreement for all these substrates except the Au film (to be discussed below) demonstrates that the dominating effect of these substrates in the single-shot ablation of TMDs is the etalon effect, even though their thermal conductivities vary over two orders of magnitudes^34^.

**Figure 4:**
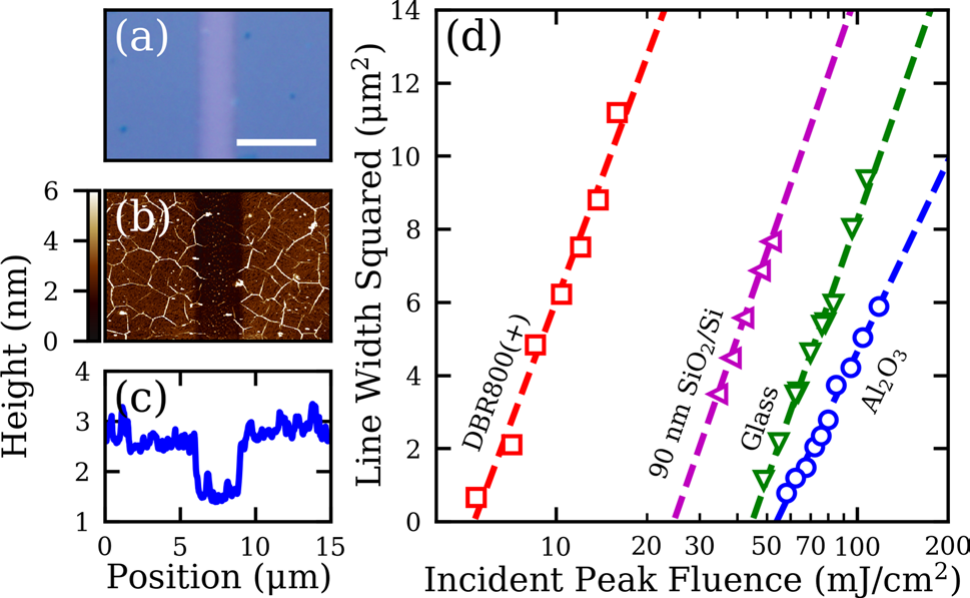
(**a**) An example OM image of a line patterned into a MoS_2_ film on 90 nm SiO_2_/Si. The scale bar is 5 µm. (**b**) The corresponding AFM height map to the OM image in (**a**). (**c**) An average line profile taken from the AFM height map in (**b**). (**d**) Plot of the line width squared versus the incident peak fluence for lines patterned in MoS_2_ on various substrates. The scan speed was set to 100 µm/s.

This result may not be too surprising, given that the total energy input for single-shot ablation is small such that substrate heating is negligible, regardless of their differences in thermal conductivities. With high-repetition-rate femtosecond lasers, however, quasi-CW laser heating of the MoS_2_ film is expected such that heat transfer to the substrates may occur during ablation (Supplementary Fig. S8). To investigate this conjecture, we conduct line-scan experiments where the MoS_2_ film is exposed to an 80 MHz pulse train from an ultrafast oscillator while translating at a constant speed. Figures 4a–c show respectively an optical microscope (OM) image, AFM height, and AFM cross-sectional profile of a line scan with a fluence of 34 mJ/cm^2^ and a scan speed of 100 μm/s on the 90 nm SiO_2_/Si substrate. Here, clean removal of monolayer MoS_2_ is also observed.

Similar to the single-shot trials in Fig. 2c, a line-scan ablation threshold $F_{th}$ for the MoS_2_ film can be extracted by extrapolating the dependence of the line width squared on the peak incident fluence. Figure 4d shows the data and the fits for various substrates, taken with a fixed scan rate of 100 μm/s and a focused laser spot radius of 2.0 µm. The extracted line-scan $F_{th}$ of MoS_2_ are 54, 49, 25, and 5 mJ/cm^2^ for Al_2_O_3_, glass, 90 nm SiO_2_/Si, and DBR800(+) substrates, respectively. Analogous to the single-shot thresholds, the line-scan thresholds are fitted to Eq. (4) (Supplementary Fig. S8). This fit yields $F_{th}^{int} \approx 26\: {\text{mJ}}/{\text{cm}}^{2}$ for monolayer MoS_2_ at a scanning speed of 100 μm/s. The normalized thresholds $F_{th}^{\prime}$ for the line-scan trials are then added to Fig. 3, exhibiting again excellent agreement with Eq. (5). Given the thermal nature of the quasi-CW excitation, the variation of line-scan $F_{th}$ is still largely governed by the etalon effect of the substrates. We conclude that these substrates behave as very poor heat sinks for the ultrafast laser ablation of 2D materials, irrespective of the substrates’ thermal properties. In other words, the ablation process is adiabatic with respect to the substrate in which there is negligible heat transfer between the MoS_2_ monolayer and the substrate. We attribute this adiabaticity to the very low thermal boundary conductance (TBC) between MoS_2_ and the substrates. Literature has reported TBC values ranging between 0.1 and 34 MW/m^2^/K for MoS_2_ on SiO_2_/Si substrates^35,36^ and between 19 and 38 MW/m^2^/K on a sapphire substrate^37^. Additionally, mechanically exfoliated and as-grown MoS_2_ monolayers on a SiO_2_/Si substrate are shown to have similar TBC values^36^. Therefore, we expect that ultrafast ablation of as-grown films share the same adiabaticity as the transferred films.

Our finding that the femtosecond ablation is adiabatic with respect to the substrate is in sharp contrast to multiple reports that the substrates serve as a heat sink for the laser processing of 2D materials^19,22,27,38^. For example, Yoo et al. reported $F_{th}$ = 98 mJ/cm^2^ for graphene on 285 nm SiO_2_/Si and $F_{th} <$ 43 mJ/cm^2^ for suspended graphene in single-shot femtosecond laser ablation^22^. They attributed this difference to the adiabatic condition of suspended graphene where heat dissipation through the substrate is forbidden. Based on our finding here, we offer an alternative interpretation. Considering the etalon effect, $\xi_{ZTA}$ are 0.2 and 1 for 285 nm SiO_2_/Si (see Supplementary Fig. S1d) and air substrates, respectively. Based on $F_{th}$ = 98 mJ/cm^2^ for graphene on 285 nm SiO_2_/Si substrate, we can estimate $F_{th}$ ~ 20 mJ/cm^2^ for suspended graphene, which is consistent with $F_{th}$ $<$ 43 mJ/cm^2^ reported by the authors. Moreover, the knowledge of $F_{th}^{int}$ and $\xi_{ZTA}$ (*i.e.*, Fig. 1a) makes $F_{th}$ predictable for any substrate, according to Eq. (5). For example, given that $F_{th} = 54\: {\text{mJ}}/{\text{cm}}^{2}$ (Fig. 2c) and $\xi_{ZTA} = 1.14$ (Fig. 1a) for the 90 nm SiO_2_/Si substrate, the predicted threshold for the DBR800(+) substrate with $\xi_{ZTA} = 3.97$ (Supplementary Fig. S1e) is $F_{th} = 15\: {\text{mJ}}/{\text{cm}}^{2}$, which matches very well with the experimental threshold of 16 mJ/cm^2^.

**Figure 5:**
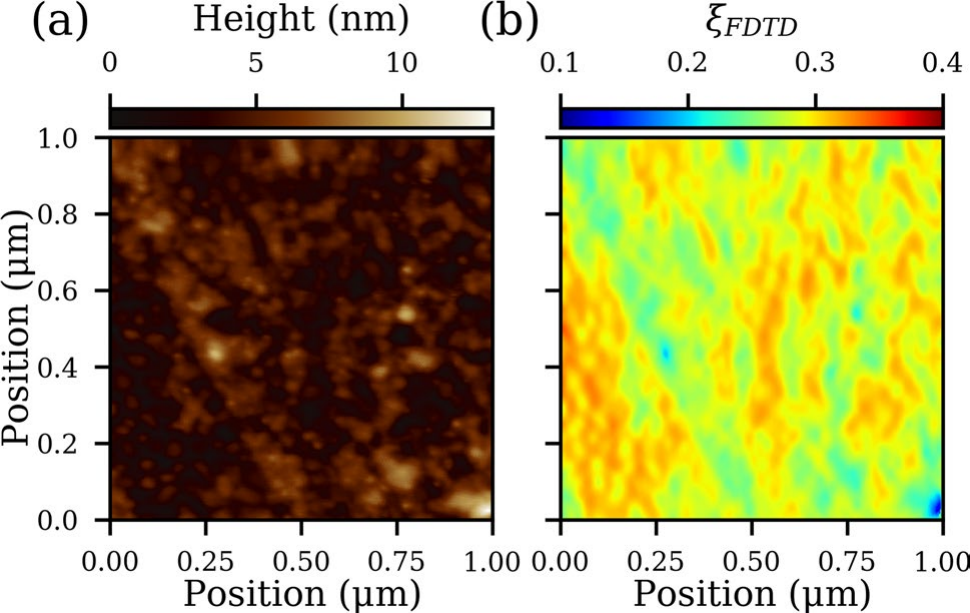
(**a**) AFM height scan of a 1 μm × 1 μm square of the Au surface. (**b**) Calculated intensity enhancement $\xi_{FDTD} { }$ across the simulation surface based on the AFM image in (**a**). See the text for details.

Among the single-shot trials (solid circles) in Fig. 3, the predicted ablation threshold based on $\xi_{ZTA}$ for a smooth Au film is 40% higher than the experimental value, indicating the presence of an additional enhancement process beyond the etalon effect that increases the internal field. An AFM measurement (Fig. 5a) revealed that the Au film substrate has a peak-to-peak surface roughness of 13 nm and an RMS value of 1.54 nm. This rough Au surface could lead to a local plasmonic enhancement of the incident field. Figure 5b shows a FDTD simulation of the electric field distribution at a fixed height of 0.325 nm (corresponding to half of the monolayer thickness) above the maximum height in Fig. 5a. The result is only approximate, as the MoS_2_ film may conform to the Au surface which is unaccounted in the current simulation. Additionally, the MoS_2_ film itself is not included in the simulation to ease the computational demand and to comply with the ZTA. Nevertheless, the laterally averaged intensity enhancement factor in Fig. 5b yields a much better match with $F_{th}^{\prime}$ for the Au substrate, as indicated by the empty circle in Fig. 3. More importantly, this result demonstrates that plasmonically active substrates could also be used to enhance the ablation of 2D materials compared to a flat metal surface. With a stronger plasmonically active substrate, even larger enhancements would be possible to further increase the ablation efficiency.

### Ultrafast laser patterning

**Figure 6:**
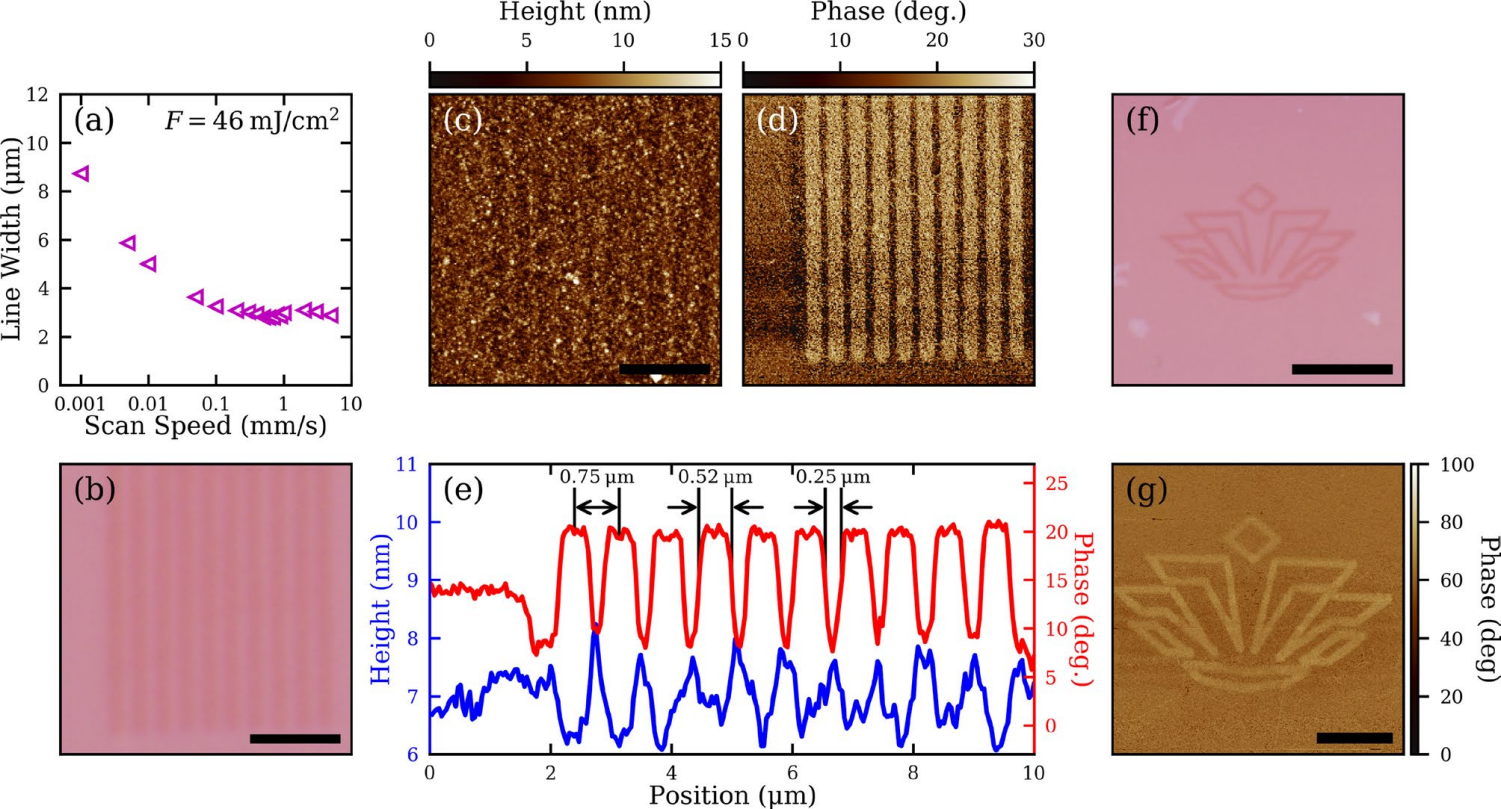
(**a**) A plot of the patterned linewidth in a MoS_2_ film on a 90 nm SiO_2_/Si substrate as a function of the scan speed. (**b**) OM image of parallel channels patterned in MoS_2_ on the DBR800(+) substrate. The scale bar is 3 μm. The incident fluence was 10 mJ/cm^2^ and the scan rate was 5 µm/s. (**c**) AFM height and (**d**) phase maps corresponding to the OM image in (**b**). The scale bar is 3 μm. (**e**) Averaged cross-sectional profiles of the AFM height and phase maps in (**c**) and (**d**). (**f**) OM image of the UNC Charlotte crown logo patterned into a monolayer MoS_2_ film on the DBR800(+) substrate. The scale bar is 10 μm. The incident fluence was 10 mJ/cm^2^ and the scan rate was 3 µm/s. (**g**) AFM phase map of the patterned UNC Charlotte crown in (**f**). The scale bar is 5 μm.

For laser patterning applications, the patterning speed and resolution are important performance metrics. Given that SiO_2_/Si substrates are commonly used for field-effect transistors, Fig. 6a shows the ablated line width in MoS_2_ on the 90 nm SiO_2_/Si substrate as a function of the scan rate with a constant fluence of about 46 mJ/cm^2^ and a 0.26 NA focusing objective^10^. Selected OM images of ablated lines are shown in Supplementary Fig. S9. As the scan rate increases from 1 µm/s, the line width decreases from 8.7 µm before leveling off at 2.9 µm at 5 mm/s. The leveling off at high scan rates is due to the mechanical instability of the translation stage used here, where the stage vibrates resulting in larger widths and uneven lines (Supplementary Fig. S9). Nevertheless, Fig. 6a clearly demonstrates high-speed line patterning of TMDs. This translates into increased patterning efficiency of an ultrafast source compared to a CW source: with a scan rate of 5 mm/s, material can be removed at a rate greater than 14,000 µm^2^/s by ultrafast lasers, whereas CW laser thinning can only pattern monolayers at a rate of 8 μm^2^/min^19^.

To demonstrate sub-micron patterning resolution, Figs. 6b-e shows an array of ablated lines in a MoS_2_ film on the DBR800(+) substrate obtained with a laser spot diameter of ~1.3 µm using a 50× , 0.55 NA focusing objective. The AFM height image has poor quality due to the surface roughness of the DBR800(+) substrate (Supplementary Fig. S6), while the AFM phase image clearly resolves the grating pattern where an average trench width of 0.52 µm and ribbon width of 0.25 µm are measured. To demonstrate laser micro-patterning, the UNC Charlotte crown logo was patterned into a MoS_2_ film on the DBR800(+) substrate as shown in Fig. 6f. The total size of the pattern is 20 μm and was engraved using a fluence of 10 mJ/cm^2^ and a low feed rate of 3 μm/s to avoid skewing the pattern (Supplementary Fig. S9). The thicknesses of the lines in the logo were found to be around 0.7 µm as measured by the AFM phase mapping. For practical applications, cost is also an important consideration. Although Figs. 2c and 4d have demonstrated femtosecond ablation and patterning of MoS_2_ on several substrates, the large field enhancement of the DBR800(+) substrate only requires pulse energies as low as 1 nJ for single-shot ablation and on the order of 100 pJ for line scans, as demonstrated in Fig. 6. This pulse energy translates to an average power of 80 mW which is readily available from compact femtosecond oscillators (Supplementary Fig. S9). With a proper design, the substrate could be engineered to enhance both the patterning process and the light-coupling performance of the resulting device. Alternatively, the patterned film can be transferred to other substrates^20,39^.

## Conclusion

In conclusion, femtosecond laser patterning of monolayer MoS_2_ was performed for the first time, where we demonstrated scan rates as high as 5 mm/s and resolutions as low as 250 nm under modest focusing conditions. We observed a nearly 20× variation in the threshold fluence for the femtosecond ablation of transferred MoS_2_ monolayers on several substrates. This variation is attributed to the etalon effect where the substrate modulates the internal light intensity within the monolayer. An intrinsic ablation threshold $F_{th}^{int}$ is thereby introduced as a substrate-independent threshold parameter for the laser ablation of 2D materials, which were found to be 66 mJ/cm^2^ and 26 mJ/cm^2^ for single-shot and quasi-CW ablation (80 MHz pulse train at a scanning speed 100 μm/s), respectively, for MoS_2_. With this knowledge, we showed that the incident threshold fluence on any substrate is easily predicted. Additionally, we proved that the ablation process is adiabatic with respect to the substrate due to the very poor thermal boundary conductance between the monolayer and the substrates, which contradicts the common view that substrates serve as heat sink for laser processing. Importantly, we also introduced the zero-thickness approximation for quick and accurate estimation of the etalon effect in monolayers, which is shown to be independent of the 2D materials and applicable for any optical excitation of 2D materials beyond laser ablation. Furthermore, substrate engineering is demonstrated to enhance the ablation efficiency by 7× , enabling future patterning of 2D materials with low-power oscillators. Finally, the notion of the intrinsic threshold fluence highlights the importance of invoking the internal field instead of the incident field for studying strong-field phenomena in monolayers, including nonlinear absorption, saturable absorption, dielectric breakdown, etc., which, as it stands, also have significantly conflicting reported values, largely because they all neglect the etalon effect in their analysis^40,41^. Although transferred MoS_2_ monolayers were studied in this work, we expect our findings can be generalized to other 2D materials, both transferred and as-grown. Our work elucidates the role of substrates and firmly establishes femtosecond laser ablation as a viable route to pattern 2D materials.

## Methods

### Sample preparation

Highly-oriented, monolayer MoS_2_ films were grown by CVD on Al_2_O_3_ following the procedure outlined in reference 42^42^. Monolayer growth was confirmed by atomic force microscopy and photoluminescence and Raman spectroscopy (Supplementary Fig. S4). All films were transferred to their host substrates which included 70 nm Au film, Al_2_O_3_, borosilicate glass, 90 nm SiO_2_/Si, and two different DBR substrates. The transfer process is also outlined in reference 42^42^.

### Single-shot experiments

A Coherent RegA 9000 operating at 800 nm with a pulse duration of 160 fs at the sample surface was used for all single-shot experiments. The laser was operated at a repetition rate of 307 Hz and a mechanical shutter was used to select out single pulses. Each spot on the film was only exposed to a single pulse in order to avoid incubation effects. The pulse energy was recorded with a calibrated photodiode. Optical images of the ablation features were captured using an Olympus BX51 optical microscope. The ablation areas were measured with the software ImageJ. A minimum of five ablation features were made per pulse energy and averaged for analysis.

### Line-scan and laser-patterning experiments

A Spectra-Physics Tsunami operating at 800 nm with a pulse duration of 210 fs and a repetition rate of 80 MHz was used for all line-scan and laser-patterning experiments. Sample translation and positioning was performed using an Aerotech ANT three-axis motorized translation stage. The pulse energy was simultaneously recorded using a calibrated photodiode.

### FDTD simulations

FDTD simulations were carried out using Lumerical. The simulation space was 1.3 μm × 1.3 μm and consisted of a plane wave (TFSF source) at normal incidence with dimensions of 1.05 μm × 1.05 μm to illuminate the entire Au surface area which was 1.0 μm × 1.0 μm. The mesh size for the rough Au surface was 2 × 2 × 0.4 nm while the mesh step size for propagation into the bulk Au film was 5 nm. The field strength was monitored in a 1 μm × 1 μm square located 0.325 nm above maximum point of the Au surface.

## Supplementary Information

Supplementary Information.

## Data Availability

The data supporting the conclusions is all contained within the manuscript and supplementary information.
