# Supplementary material for: Ultrafast laser ablation, intrinsic threshold, and nanopatterning of monolayer molybdenum disulfide
Source: Sci Rep. 2022 Apr 28;12:6910. doi: 10.1038/s41598-022-10820-w (PMC9050692; doi:10.1038/s41598-022-10820-w)
Supplement: Supplementary file 1 — Supplementary Information. [file 41598_2022_10820_MOESM1_ESM.pdf]

Supplementary Information:
Ultrafast Laser Ablation, Intrinsic Threshold, and Nanopatterning of Monolayer Molybdenum Disulfide

Joel M. Solomon[1], Sabeeh Irfan Ahmad[1], Arpit Dave[1], Li-Syuan Lu[2,3], Fatemeh HadavandMirzaee[1], Shih-Chu Lin[2], Sih-Hua Chen[2], Chih-Wei Luo[2,4,5], Wen-Hao Chang[2,3], and Tsing-Hua Her[1,*]

[1] Department of Physics and Optical Science, The University of North Carolina at Charlotte, Charlotte, North Carolina 28223, United States
[2] Department of Electrophysics, National Yang Ming Chiao Tung University, Hsinchu 30010, Taiwan
[3] Research Center for Applied Sciences, Academia Sinica, Taipei 11529, Taiwan
[4] Institute of Physics and Center for Emergent Functional Matter Science, National Yang Ming Chiao Tung University, Hsinchu 30010, Taiwan
[5] National Synchrotron Radiation Research Center (NSRRC), Hsinchu 30076, Taiwan
[*] ther@uncc.edu

Calculation of Internal Field in 2D Materials

When light is incident on 2D materials (2DMs) such as graphene, hexagonal boron nitride, or transition metal dichalcogenides (TMDs), the field inside the 2DM can be enhanced or attenuated due to the etalon effect between the air, 2DM, and substrate. The system can be modeled as an asymmetric etalon composed of air (refractive index $\tilde{n}_0$), 2DM ($\tilde{n}_1$), and a substrate ($\tilde{n}_s$) (Fig. S1a). By considering a normally incident field $\mathcal{E}_{inc}$ and their multiple reflections from two interfaces, the electric field $\mathcal{E}_{2DM}$ at a point x within the 2DM follows the standard Airy formula to be

$$\mathcal{E}_{2DM}(x) = \mathcal{E}_{inc}\tilde{t}_{01}\left(\frac{e^{i\beta_1 x} + \tilde{r}_{1s}e^{i\beta_1(2d_1 - x)}}{1 - \tilde{r}_{1s}\tilde{r}_{10}e^{i2\beta_1 d_1}}\right), \tag{S1}$$

where $\beta_j = 2\pi\tilde{n}_j/\lambda_0$, d_1 is the thickness of the 2DM, $\tilde{t}_{01} = 2/(1+\tilde{n}_1)$ and $\tilde{r}_{10} = (\tilde{n}_1 - 1)/(\tilde{n}_1 + 1)$ are Fresnel coefficients, and $\tilde{r}_{1s}$ is the effective reflection coefficient of the substrate, as seen from the 2DM. The internal intensity quoted in this work is the average $|\mathcal{E}_{2DM}|^2$, calculated from Eq. (S1) by

$$|\mathcal{E}_{2DM}|^2 = \frac{1}{d_1}\int_0^{d_1} \mathcal{E}_{2DM}^*(x)\mathcal{E}_{2DM}(x)\, dx. \tag{S2}$$

Given the above equations, the zero-thickness approximation (ZTA) can be applied. Specifically, the ZTA considers the case where the wavelength λ is much greater than the thickness of the 2D material d_1. In other words, if we consider the limit $\lambda \gg d_1$, Eq. (S1) reduces to Eq. (1) in the main text.

For single-material substrates such as Al_2O_3, cover glass, or a thick Au film, $\tilde{r}_{1s}$ is simply the Fresnel reflection coefficient:

$$\tilde{r}_{1s} = \frac{\tilde{n}_1 - \tilde{n}_s}{\tilde{n}_1 + \tilde{n}_s}. \tag{S3}$$

For SiO_2/Si substrates, $\tilde{r}_{1s}$ can be calculated analytically using an asymmetric etalon composed of a TMD, SiO_2 and Si, which yields

$$\tilde{r}_{1s} = \frac{(\tilde{n}_2+\tilde{n}_s)(\tilde{n}_1-\tilde{n}_2)+(\tilde{n}_1+\tilde{n}_2)(\tilde{n}_2-\tilde{n}_s)e^{i2\beta_2 d_2}}{(\tilde{n}_2+\tilde{n}_s)(\tilde{n}_1+\tilde{n}_2)+(\tilde{n}_1-\tilde{n}_2)(\tilde{n}_2-\tilde{n}_s)e^{i2\beta_2 d_2}}, \tag{S4}$$

where d_2 is the thickness of the SiO_2 layer and $\tilde{n}_1$, $\tilde{n}_2$, and $\tilde{n}_s$ are the refractive indices of the 2DM, SiO_2, and Si, respectively. $\tilde{r}_{1s}$ as a function of the thickness of the SiO_2 layer is shown in Fig. S1c, from which the intensity enhancement factor ξ based on Eq. (S1) is shown as the blue solid line in Fig. S1d. The good agreement between ξ and ξ_{ZTA}, regardless of the thickness of the SiO_2 layer, supports the validity of the ZTA.

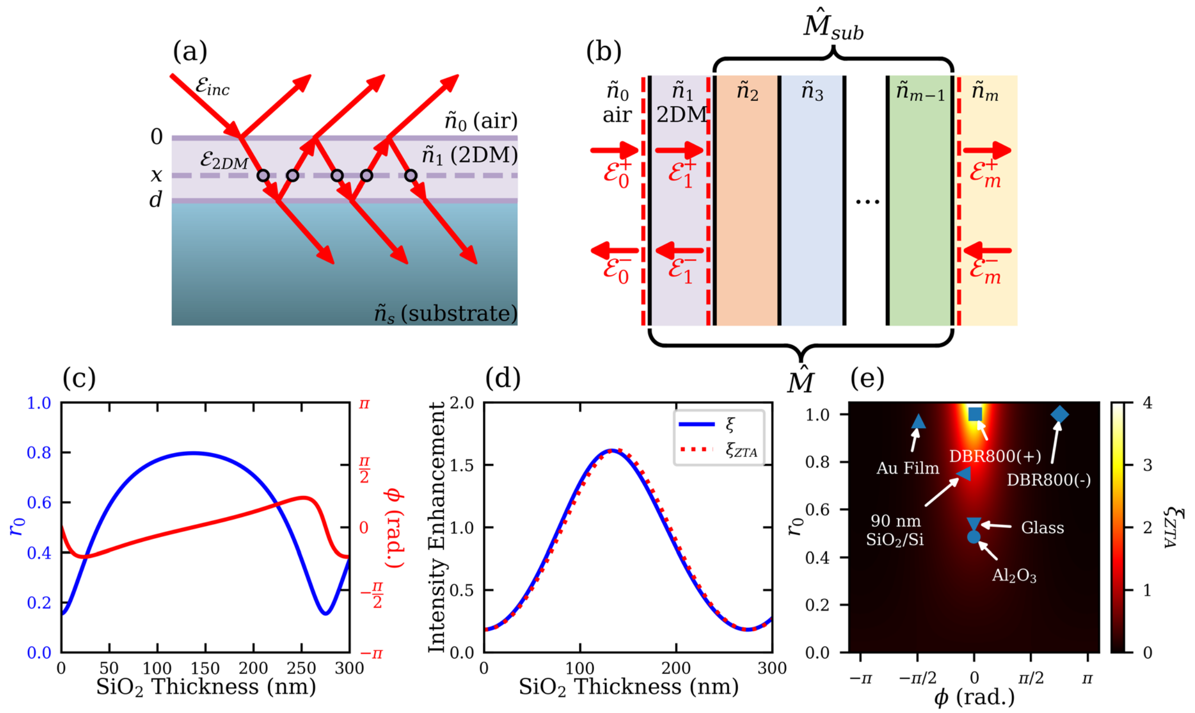

Figure S1. (a) Diagram for calculating the internal field strength in a 2D material. (b) Diagram for calculating the internal field in a 2D material on a stratified media based on a transfer matrix method. (c) The calculated effective reflection coefficient $\tilde{r}_{1s}$ between a MoS_2 and a SiO_2/Si substrate as a function of SiO_2 thickness, when excited at 800 nm. The reflection coefficient $\tilde{r}_{1s}$ is expressed as $\tilde{r}_{1s} = r_0 e^{i\phi}$ to plot in terms of its amplitude and phase. (d) The intensity enhancement factor for an MoS_2 film supported by an SiO_2/Si substrate. (e) The calculated intensity enhancement factor as a function of the effective reflection coefficient $\tilde{r}_{1s} = r_0 e^{i\phi}$. The substrates tested in the main text are marked within the plot.

For arbitrary stratified substrates, the effective reflection coefficient $\tilde{r}_{1s}$ can be calculated using a transfer matrix method (TMM). The layer indices are shown in Fig. S1b, where dashed lines indicate the position to which fields are referenced. The substrate is composed of layers 2 through m-1, which connects the input and output fields according to

$$\begin{bmatrix} \mathcal{E}_1^+ \\ \mathcal{E}_1^- \end{bmatrix} = \hat{I}_{12}\hat{T}_2\hat{I}_{23}\hat{T}_3 \cdots \hat{T}_{m-1}\hat{I}_{m-1,m} \begin{bmatrix} \mathcal{E}_m^+ \\ \mathcal{E}_m^- \end{bmatrix} = \hat{M}_{sub} \begin{bmatrix} \mathcal{E}_m^+ \\ \mathcal{E}_m^- \end{bmatrix}, \tag{S5}$$

where

$$\hat{I}_{jk} = \text{Interface Matrix} = \frac{1}{\tilde{t}_{jk}}\begin{bmatrix} 1 & \tilde{r}_{jk} \\ \tilde{r}_{jk} & 1 \end{bmatrix},$$

$$\hat{T}_j = \text{Propagation Matrix} = \begin{bmatrix} e^{-i\beta_j d_j} & 0 \\ 0 & e^{i\beta_j d_j} \end{bmatrix}, \tag{S6}$$

$$\hat{M}_{sub} = \text{Substrate Matrix} = \begin{bmatrix} M_{sub,00} & M_{sub,01} \\ M_{sub,10} & M_{sub,11} \end{bmatrix}.$$

In the above equation, $\tilde{r}_{jk}$ is the Fresnel reflection coefficient, $\tilde{t}_{jk}$ is the Fresnel transmission coefficient from the jth to k^{th} medium, and d_j is the thickness of the jth material. If the field in the final layer traveling left ($\mathcal{E}_m^-$) is zero, dividing Eq. (S5) by $\mathcal{E}_1^+$ yields the effective reflection and transmission coefficients of the composite substrate given by

$$\tilde{r}_{1s} = \frac{\mathcal{E}_1^-}{\mathcal{E}_1^+} = \frac{M_{sub,10}}{M_{sub,00}},$$

$$\tilde{t}_{1s} = \frac{\mathcal{E}_m^+}{\mathcal{E}_1^+} = \frac{1}{M_{sub,00}}. \tag{S7}$$

To prove that $\mathcal{E}_{2DM}^{ZTA}$ is independent of the 2DM for arbitrary stratified substrates, we note that the only dependence on the 2D material's refractive index is at the interface between layers 1 and 2. We then define a $\hat{S}$ matrix according to $\hat{M}_{sub} = \hat{I}_{12}\hat{S}$, and the effective reflection coefficient $\tilde{r}_{1s}$ can easily be shown to be

$$\tilde{r}_{1s} = \frac{S_{10} + \tilde{r}_{12}S_{00}}{S_{00} + \tilde{r}_{12}S_{10}}, \tag{S8}$$

where Eq. (S8) is rigorous. To prove that $\mathcal{E}_{2DM}^{ZTA}$ is independent of the 2DM, substituting Eq. (S8) into Eq. (1) of the main text gives

$$\mathcal{E}_{2DM}^{ZTA} = \mathcal{E}_{inc}\frac{2(S_{00} + S_{10})}{(1 + \tilde{n}_2)S_{00} + (1 - \tilde{n}_2)S_{10}}. \tag{S9}$$

Equation (S9) offers a general proof that the $\mathcal{E}_{2DM}^{ZTA}$ is independent of the 2D material's refractive index ($\tilde{n}_1$) for any arbitrary substrate when light is at normal incidence.

For the DBR800(+) substrate with N periodic bi-layers (Fig. S5a; even layers are SiO_2 and odd layers are TiO_2), the $\hat{S}$ matrix at normal incidence can be calculated analytically according to[1]

$$\hat{S} = \begin{bmatrix} S_{00} & S_{01} \\ S_{10} & S_{11} \end{bmatrix} = \begin{bmatrix} AU_{N-1} - U_{N-2} & BU_{N-1} \\ CU_{N-1} & DU_{N-1} - U_{N-2} \end{bmatrix} \tag{S10}$$

where

$$\begin{aligned} A &= \frac{1}{\tilde{t}_{23}\tilde{t}_{32}} e^{-i\beta_2 d_2} e^{-i\beta_3 d_3} \left(1 - \tilde{r}_{23}^2 e^{i2\beta_3 d_3}\right) \\ B &= \frac{\tilde{r}_{23}}{\tilde{t}_{23}\tilde{t}_{32}} e^{-i\beta_2 d_2} \left(e^{i\beta_3 d_3} - e^{-i\beta_3 d_3}\right) \\ C &= \frac{\tilde{r}_{23}}{\tilde{t}_{23}\tilde{t}_{32}} e^{i\beta_2 d_2} \left(e^{-i\beta_3 d_3} - e^{i\beta_3 d_3}\right) \\ D &= \frac{1}{\tilde{t}_{23}\tilde{t}_{32}} e^{i\beta_2 d_2} e^{-i\beta_3 d_3} \left(e^{i2\beta_3 d_3} - \tilde{r}_{23}^2\right) \\ U_N &= \frac{\sin\left\{(N+1)\arccos\left[\frac{1}{2}(A+D)\right]\right\}}{\sqrt{1 - \frac{1}{4}(A+D)^2}}. \end{aligned} \tag{S11}$$

The $\mathcal{E}_{2DM}^{ZTA}$ for the DBR800(+) substrate at normal incidence is therefore

$$\mathcal{E}_{2DM}^{ZTA} = \mathcal{E}_{inc} \frac{2(AU_{11} - U_{10} + CU_{11})}{(1+\tilde{n}_2)(AU_{11} - U_{10}) + (1-\tilde{n}_2)CU_{11}} \tag{S12}$$

where $N = 12$.

Limitations of the Zero-Thickness Approximation

The ZTA only applies in the condition where $\lambda \gg d_1$. For multilayer films of 2D materials, the ZTA will eventually breakdown as the number of layers increase in which case Eq. (S1) will be required to properly calculate the field strength within the 2D material. As an example, we calculated the electric field strength $\mathcal{E}_{2DM}$ as a function of layer number for MoS_2 supported by a sapphire (Al_2O_3) substrate. Specifically, we calculated the intensity enhancement factor ξ based on the ZTA, Eq. (S2) which averages (Ave) the field strength within the 2D material, and Eq. (S1) for $x = 0$ which corresponds to the field strength at the surface (Top) of the 2D material. As shown in Fig. S2, the ZTA deviates by $\gtrsim$10% for 7 or more layers. While the ZTA cannot be used for bulk MoS_2, it can still provide accurate results for 2D materials consisting of a few layers.

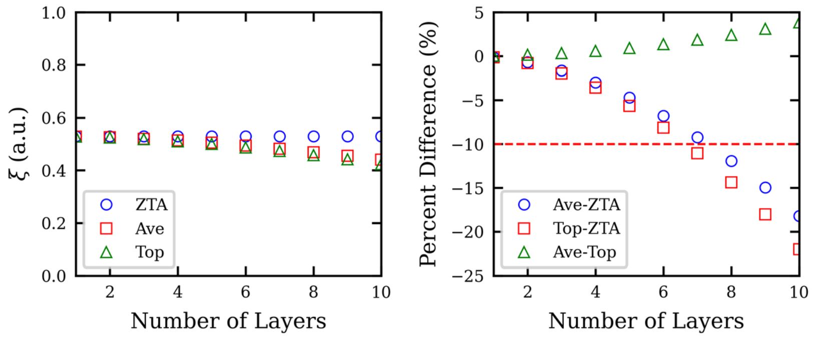

Figure S2. (left) The calculated intensity enhancement factor ξ based on the ZTA, Eq. (S2) (Ave), and Eq. (S1) for $x = 0$ (Top). (right) The percent difference in ξ calculated in the plot on the left.

MoS_2 Characterization

As described in the main text, monolayer MoS_2 films and flakes were grown by chemical vapor deposition (CVD) on *c*-cut sapphire (Al_2O_3) substrates as illustrated in Fig. S3a. The density of the flakes is determined based on the position of the substrate in the chamber. As shown in Fig. S3b-c, the end of the substrate closest to the sulfur and MoO_3 powder yields the highest density, resulting in a continuous film whereas the edge of substrate furthest from the reactants produces single crystal flakes. For the experiments conducted here, the monolayer MoS_2 film was transferred to several different substrates. An example optical image of an MoS_2 film transferred to a borosilicate glass substrate is presented in Fig. S3d where the monolayer film, bilayer islands, and the substrate are labeled. The bilayer islands were avoided for all experimental results.

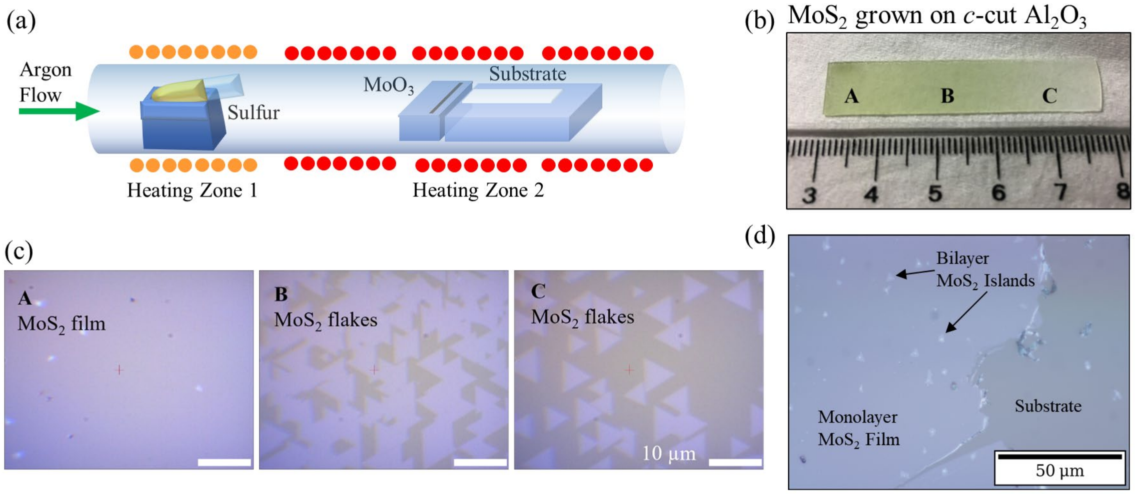

Figure S3. (a) Schematic for CVD growth of MoS_2. (b) Image of monolayer MoS_2 grown on *c*-cut Al_2O_3. (c) Optical image showing change in MoS_2 density from a continuous film in A to single crystal flakes in C. The images A, B, and C correspond to the points A, B, and C in (b). (d) Optical image of a monolayer MoS_2 film transferred to a borosilicate glass substrate.

In addition to optical microscopy, atomic force microscopy (AFM), photoluminescence spectroscopy, and Raman spectroscopy were used to confirm monolayer growth. Figure S4a shows an AFM image of a pristine MoS_2 flake grown on a sapphire substrate where the thickness was measured to be 0.63 nm, confirming that the flake is a monolayer. During the transfer process,

monolayer film and flakes may develop cracks or wrinkles as shown in the AFM image in Fig. S4b. Additionally after the transfer process, the height of the transferred MoS_2 monolayer typically lies between ~1-2 nm as shown in Fig. S4b. This height is consistent with the heights measured for a hole ablated in an MoS_2 film due to an ultrafast pulse, providing confirmation that the underlying substrate is not damaged during experiments and eliminating the possibility that damage to the MoS_2 occurs due to material ejection from the underlying substrate. As seen in Fig. S4c, photoluminescence measurements also confirm monolayer growth where strong photoluminescence emission at ~660 nm (~1.88 eV) is recorded due to the excitonic resonance. Furthermore, Raman measurements reveal that the separation between the in-plane (E_{2g}^{1}) and out-of-plane (A_{1g}) vibrational modes is 18 cm^{-1}, consistent with that of a monolayer. The separation of the two Raman modes remains 18 cm^{-1} after the MoS_2 film is transferred to a new substrate.

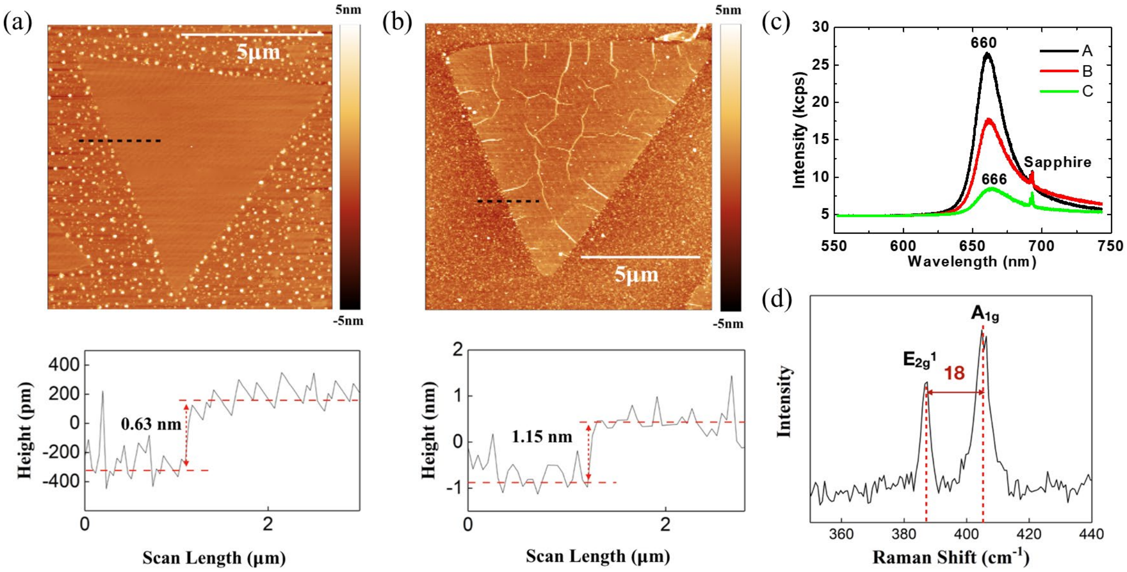

Figure S4. (a) AFM scan of an as-grown monolayer MoS_2 flake on an Al_2O_3 substrate. (b) AFM scan of a monolayer MoS_2 flake transferred to a new Al_2O_3 substrate. (c) Photoluminescence spectra of as-grown MoS_2 on Al_2O_3. The lines A, B, and C correspond to the image in Fig. S3b. (d) Raman spectra of as-grown MoS_2 on Al_2O_3.

DBR Design and Characterization

As noted in the main text, two custom designed distributed Bragg reflector (DBR) substrates are employed in this study: one of which (DBR800(+)) targets maximal intensity enhancement of 4 ($\xi_{ZTA} = 4$, $r_0 = 1$, $\phi = 0$) and the other (DBR800(-)) targets maximal intensity suppression ($\xi_{ZTA} = 0$, $r_0 = 1$, $\phi = \pi$) for 800 nm light. Both DBRs feature multiple quarter-wave stacks of a low index material SiO_2 ($n = 1.45$) and a high index material TiO_2 ($n = 2.08$) for a center wavelength of 800 nm. The design of the DBR800(+) is shown in Fig. S5a, which contains 12 pairs of stacks including the SiO_2 as the terminating layer to produce a zero-phase shift upon reflection for total constructive interference within the 2D material. The design of the DBR800(-) is shown in Fig. S5d, which contains 11 pairs of stacks plus additional TiO_2 as the terminating layer to produce a π-phase shift upon reflection for total destructive interference within the 2D material.

To account for manufacturing imperfections in thickness control of the DBR substrates, their reflectivities were measured and fitted to their theoretical design to extract actual layer thicknesses (Figs. S5b and S5e). These extracted values are then used to calculate the theoretical reflectivity and phases (Figs. S5c and S5f) and the effective reflection coefficients of the DBR substrates using Eq. (S8), which yield $r_0 = 1.0$, $\phi = 0.011\pi$, and $r_0 = 1.0$, $\phi = 0.76\pi$ for the DBR800(+) and DBR800(-), respectively. Measuring the actual layer thicknesses is important to best model the DBR's performance and to predict the actual field enhancement or attenuation experienced by the 2D material.

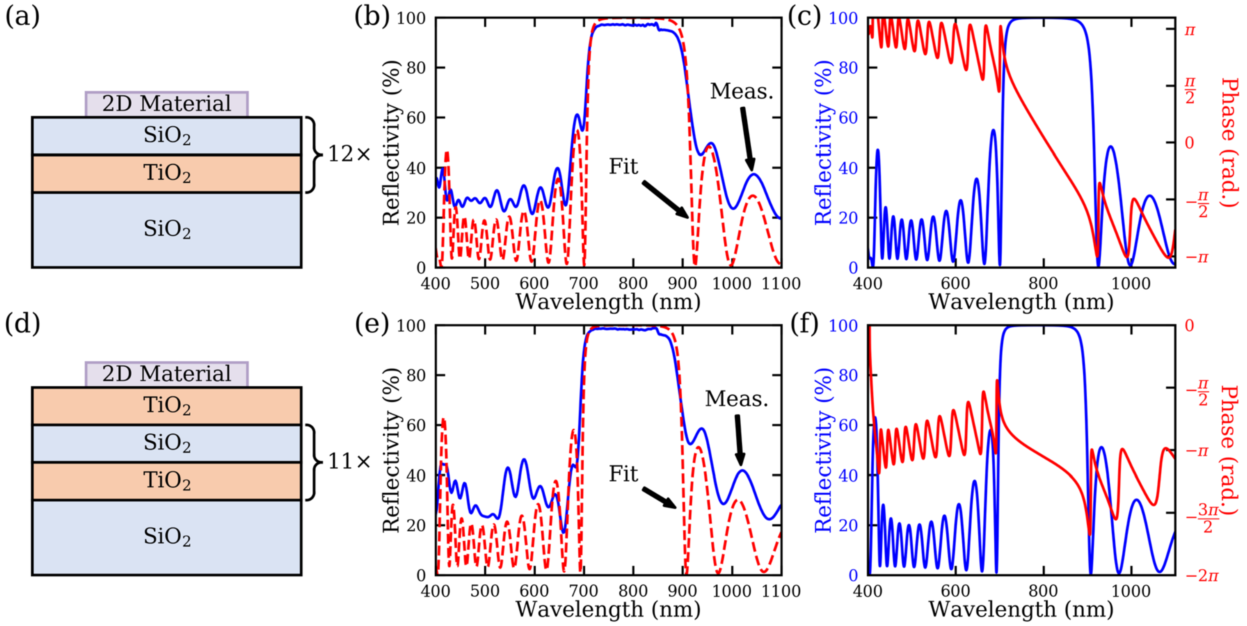

Figure S5. (a) Design for the DBR800(+) substrate. (b) Measured and fitted reflectivity for the DBR800(+). (c) Calculated reflectivity and phase for the DBR800(+). (d) Design for the DBR800(-) substrate. (e) Measured and fitted reflectivity for the DBR800(-). (f) Calculated reflectivity and phase for the DBR800(-).

As part of the DBR and MoS_2 film characterization, AFM scans of the surface were taken. Figure S6a shows an AFM height map of the edge of the MoS_2 film on the DBR800(+) substrate. The height of the monolayer is difficult to resolve due to the surface roughness of the DBR substrate itself. The MoS_2 film can be clearly resolved using the phase mapping capability of the AFM instrument as shown in Fig. S6b. A significant phase change is measured when the probe is tapping the DBR surface versus the MoS_2 film. This capability allows one to identify when the material properties on the surface has changed. Although the DBR surface was not smooth, the surface roughness had negligible impact on the surface field enhancement as seen in the good agreement between the experimental results and the TMM calculations (Fig. 3 in the main text).

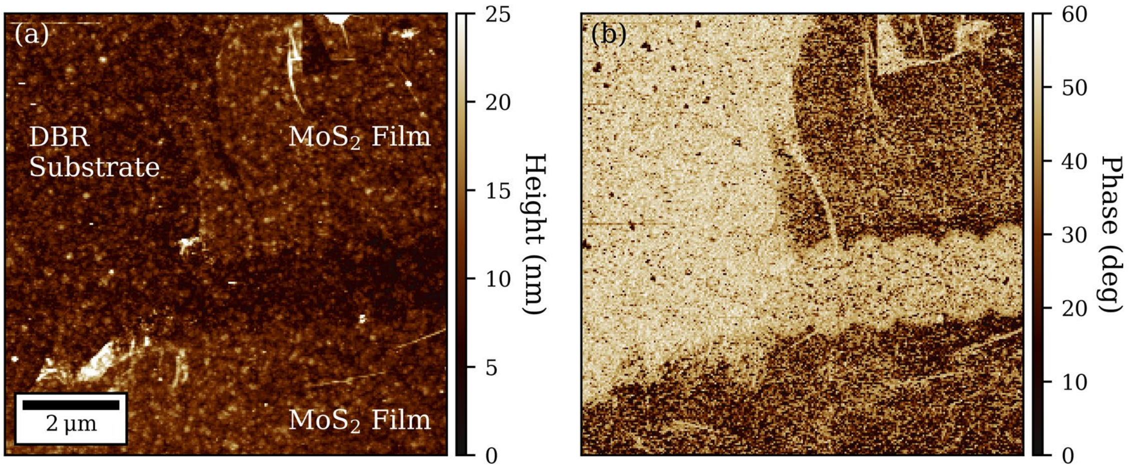

Figure S6. (a) AFM height map of monolayer MoS_2 supported by the DBR800(+) substrate. (b) AFM phase map for the same region as in (a).

Determination of Focused Laser Spot Size, Threshold Fluence, and Intrinsic Threshold Fluence

For a Gaussian laser spot, the diameter D of an ablation feature is given by[2]

$$D_{u,v}^2 = 2w_{u,v}^2 \ln\left(\frac{E}{E_{th}}\right) = 2w_{u,v}^2 \ln\left(\frac{F}{F_{th}}\right) \tag{S13}$$

where w is the laser spot radius at an intensity e^{-2}, E is the pulse energy, E_{th} is the pulse energy at the ablation threshold, F is the peak fluence of the pulse, and F_{th} is the peak fluence at threshold. The subscripts u and v represent the major and minor axes, respectively, of the laser spot profile. The peak fluence for a pulse with a Gaussian spatial and temporal profile is given by

$$F = \frac{2E}{\pi w_u w_v} = \frac{2E}{\pi w_{eff}^2} = 2F_{ave} \tag{S14}$$

where $w_{eff} = \sqrt{w_u w_v}$ is the effective laser spot radius and F_{ave} is the average laser fluence. Equation (S13) consists of two expressions for pulses with elliptical spatial profiles; however, the two equations can be combined in terms of the ablation area giving

$$A = \frac{\pi}{2} w_{eff}^2 \ln\left(\frac{E}{E_{th}}\right) = \frac{\pi}{2} w_{eff}^2 \ln\left(\frac{F}{F_{th}}\right), \tag{S15}$$

where A is the ablation area. This relationship also allows the determination of both the laser spot size and ablation threshold *in situ*. The effective e^{-2}-intensity beam radius determined from the fits were found to be 1.9 μm for the single-shot ablation trials, which agrees with the spot measured by a beam profiler within 5%. This result suggests there is little or no lateral carrier diffusion in monolayer MoS_2 during the time scale of the ablation. Examples of the fits for Eq. (S13) and (S15) are demonstrated in Fig. S7 for the ablation of monolayer MoS_2 on borosilicate glass.

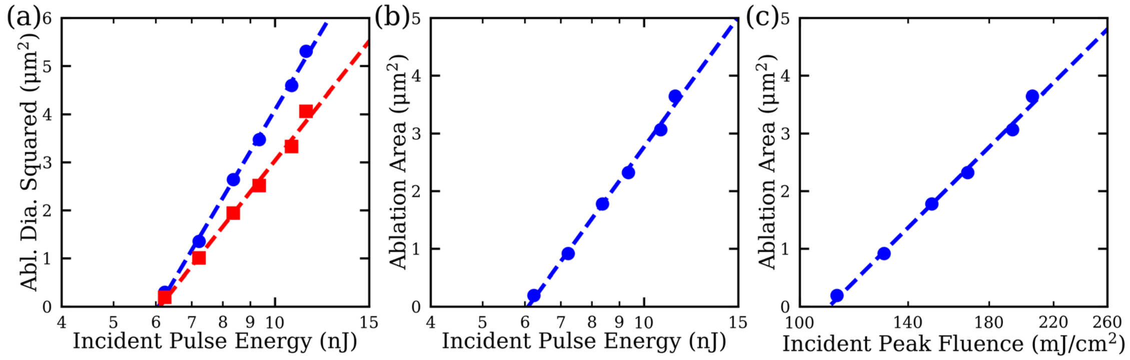

Figure S7. (a) Ablation diameters for MoS_2 on a borosilicate glass cover slip. The major and minor axes are fitted to Eq. (S13). (b) Ablation area for MoS_2 as a function of pulse energy with its fit to Eq. (S15). (c) Ablation area of MoS_2 as a function of the peak fluence of the incident pulse with its fit to Eq. (S15).

When determining the damage threshold with the oscillator as shown in Fig. 4d of the main text, the line width is fitted to Eq. (S13) to extract the threshold. Although the experimental data follows the same logarithmic dependence on the fluence, care needs to be taken when extracting out the spot size. Equations (S13) and (S15) were derived under the assumption that lateral carrier diffusion is negligible. For line scans with an oscillator, material modification or breakdown is dominated by thermal mechanisms where lateral carrier diffusion may not be negligible. For example, at a peak fluence of 34 mJ/cm^2 and a scan rate of 100 μm/s (Fig. 4a-c in the main text), the pulse energy is 2.2 nJ and the separation between adjacent pulses is only 1.25 pm. Given a translation distance of 2.0 μm which is equal to the focused laser spot radius, the MoS_2 film will have been exposed to ~1.6 million pulses and a total energy of ~3.5 mJ. This energy is several orders of magnitude greater than the maximum 22 nJ pulses used in the single-shot ablation experiments. Consequently, thermal accumulation effects are expected to play a significant role in the line-scan experiments. Additionally, line scans only scan across one axis of the laser spot which was found to be slightly elliptical and suffered from astigmatism. Accordingly, the slope of the data in Fig. 4d depends on whether the scan direction was along the

minor or major axis of the laser spot. Due to these two reasons, the effective laser spot radius was determined using a CCD camera and was found to be 2.0 μm for the oscillator.

After extracting the ablation thresholds with Eq. (S13) and (S15) for the line scan and single shot experiments, respectively, the intrinsic ablation threshold was determined by fitting the thresholds to Eq. (4) in the main text. These fits are shown in Fig. S8.

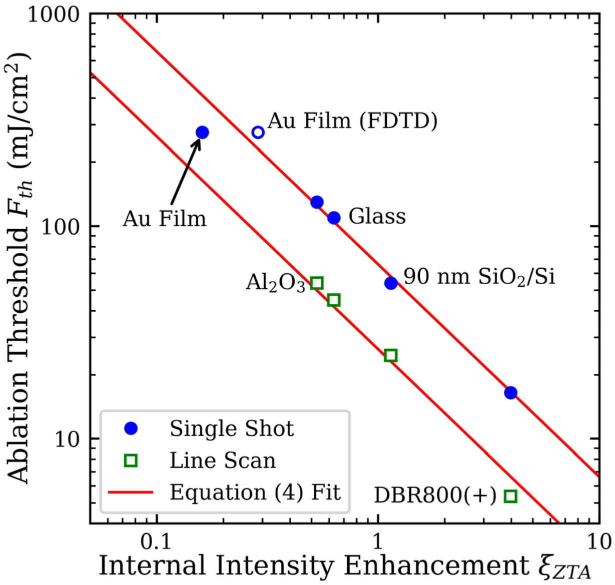

Figure S8. Determination of the intrinsic ablation threshold F_{th}^{int} for the single shot and oscillator-based line scan experiments. For the single shot experiments, $F_{th}^{int} = 66\ \mathrm{mJ/cm^2}$. For the line scans with an 80 MHz oscillator, the scan rate was set to 0.1 mm/s and $F_{th}^{int} = 26\ \mathrm{mJ/cm^2}$.

Ultrafast Oscillator Laser Patterning

As mentioned in the main text, laser line scans and patterning were limited by the performance of the three-axis stage used in this work. At large scan speeds, mechanical vibrations would skew lines and widen linewidths as shown in Fig. S9 for scan rates greater than 1 mm/s. For the UNCC logo in Fig. 6f, scan rates larger than 3 μm/s would overshoot sharp corners due the size of the logo, skewing the pattern. For the features created here, the speed was ultimately limited by the three-axis translation stage used. Galvo scanners can overcome these imitations and offer improved patterning rates and efficiencies where scan speeds reaching up 25 m/s are possible[3].

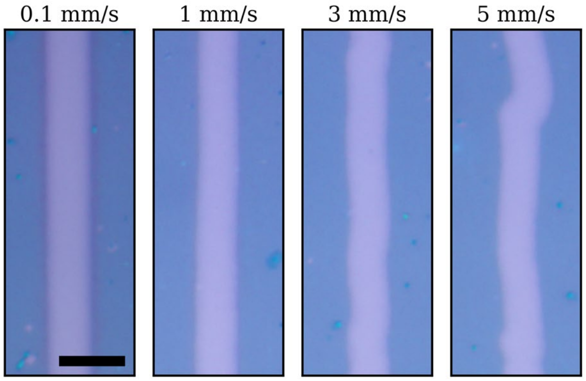

Figure S9. OM images of lines patterned into a MoS_2 film supported by a 90 nm SiO_2/Si substrate. The fluence was set at 46 mJ/cm^2. The scan rates are 0.1, 1, 3, and 5 mm/s from left to right. All images are set to the same scale with the width of the scale bar being 5 μm.

Ideally, ultrafast laser patterning of 2DMs would be conducted using oscillators due to their small form-factor and reduced cost compared to amplifiers. Fiber oscillators are especially attractive due to their superior robustness, stability, and overall ease-of-use versus their free-space counterparts. To that end, we designed the DBR800(+) substrate in order to reduce the ablation threshold of 2DMs as low as possible such that laser patterning with a commercial off-the-shelf fiber oscillator would be possible. For monolayer MoS_2 on the DBR800(+) substrate, single-shot

ablation was obtainable with 1 nJ pulse energies and laser patterning with a scan rate of 0.1 mm/s can be done with pulse energies as low as 0.37 nJ when using a 10x objective. The UNCC logo in Fig. 6f in the main text was patterned with a pulse energy of 65 pJ when the pulses were focused down with a 50x objective. All-fiber, ultrafast oscillators such as erbium-doped oscillators operating at 780 nm or yitterbium-doped oscillators operating at 1030 nm typically only produce pulses that reach up to a couple of nJ. With the DBR800(+) substrate, laser patterning with these types of systems is possible. Additionally, with recent progress made in the synthesis of wafer-scale, highly-oriented MoS_2 films, this example further demonstrates the potential for rapid production of MoS_2-based devices using ultrafast laser patterning[4]. Experimentally, a high-power Ti:S oscillator (Spectra Physics Tsunami) capable of producing 10 nJ pulses at the sample was used for patterning purposes. These larger pulse energies were needed to pattern MoS_2 films supported by the Al_2O_3 and glass substrates.

References

1 Yeh, P., Yariv, A. & Hong, C.-S. Electromagnetic propagation in periodic stratified media. I. General theory*. *J. Opt. Soc. Am.* **67**, 423-438, doi:10.1364/JOSA.67.000423 (1977).

2 Liu, J. M. Simple technique for measurements of pulsed Gaussian-beam spot sizes. *Optics Letters* **7**, 196--198, doi:10.1364/OL.7.000196 (1982).

3 Jaeggi, B., Neuenschwander, B., Zimmermann, M., Zecherle, M. & Boeckler, E. W. *Time-optimized laser micro machining by using a new high dynamic and high precision galvo scanner*. Vol. 9735 PWL (SPIE, 2016).

4 Yu, H. *et al.* Wafer-Scale Growth and Transfer of Highly-Oriented Monolayer MoS2 Continuous Films. *ACS Nano* **11**, 12001--12007, doi:10.1021/acsnano.7b03819 (2017).
